# Using HEART2 score to risk stratify chest pain patients in the Emergency Department: an observational study

**DOI:** 10.1186/s12872-022-02528-6

**Published:** 2022-03-04

**Authors:** Chet D. Schrader, Darren Kumar, Yuan Zhou, Stefan Meyering, Nicholas Saltarelli, Naomi Alanis, Chukwuagozie Iloma, Rebecca Smiley, Hao Wang

**Affiliations:** 1 Department of Emergency Medicine, John Peter Smith Health Network (JPS Health Network), 1500 S. Main St., Fort Worth, TX 76104 USA; 2 Department of Cardiology, JPS Health Network, 1500 S. Main St., Fort Worth, TX 76104 USA; 3 Department of Industrial, Manufacturing, and Systems Engineering, The University of Texas at Arlington, 701 S. Nedderman Dr., Arlington, TX 76019 USA

**Keywords:** Chest pain, Emergency Department, Cardiac imaging test, MACE

## Abstract

**Background:**

A significant number of chest pain patients had previous cardiac imaging tests (CIT) performed before being presented to the Emergency Department (ED). The HEART (history, electrocardiogram, age, risk factors, and troponin) score has been used to risk-stratify chest pain patients in the ED, but not particularly for patients with CIT performed. We aim to modify the current HEART score with the addition of most recent CIT findings (referred to as HEART2 score), to predict a 30-day major adverse cardiac event (MACE) among ED chest pain patients, compare the performance accuracy of using HEART versus HEART2 score for 30-day MACE outcome predictions, and further determine the value of HEART2 in a subset group of ED chest pain patients (i.e., ones with previous CIT).

**Methods:**

This is a single-center observational study. We included chest pain patients with HEART scores calculated during their index ED visits. A modified HEART2 score was developed with the addition of CIT findings as one of the HEART2 components. Patients were divided into three groups, including low (≤ 3), moderate (4–6), and high-risk HEART/HEART2 scores (≥ 7). MACE occurrence of a patient with different risks of HEART and HEART2 scores and overall performance accuracy of HEART versus HEART2 score predicting MACE outcomes were compared.

**Results:**

We included a total of 9419 chest pain patients at ED, among which one out of five patients (1874/9419) had previous CIT performed. Fewer (38.2%) of such patients had low-risk HEART scores in comparison to 55.5% of low-risk HEART2 scores (p < 0.001). The MACE outcomes were similar in low-risk HEART patients compared with low-risk HEART2 patients (2.2% versus 3.1%, p = 0.3021). The overall performance accuracy of using the HEART2 score to stratify chest pain patients with previous CIT was better than using the HEART score’s (AUC 0.74 versus 0.71, p = 0.0082).

**Conclusions:**

Using the HEART2 score might be suitable to stratify low-to-moderate risk chest pain patients at ED with a similar 30-days MACE occurrence compared to the HEART score. More importantly, with the use of similar low-risk criteria (HEART2 ≤ 3), over 45% more chest pain patients with previous CIT performed could be discharged directly from ED.

**Supplementary Information:**

The online version contains supplementary material available at 10.1186/s12872-022-02528-6.

## Introduction

### Background

The HEART (history, electrocardiogram, age, risk factors, and troponin) score has been used widely to risk-stratify chest pain patients in the Emergency Department (ED). It has been well validated in many studies [1–3]. Chest pain patients with low HEART scores (0–3) tend to have fewer major adverse cardiac events (MACE) [4, 5]. Therefore, it is recommended that such patients can be safely discharged from ED. Previous studies found that hospital admissions have been reduced by greater than 15% using the HEART score without increasing MACE among ED chest pain patients [6, 7]. On the other hand, chest pain patients who were considered high-risk ACS due to higher HEART scores were admitted to the hospitals. Such patients, once hospitalized, 20%-80% of which underwent cardiac imaging tests (CIT), with the majority yielding negative results [8–10]. Even with recent negative CIT findings, recurrent chest pain patients still result in higher rehospitalizations. This raises questions about the necessity of rehospitalization and the value of the previous CIT among these recurrent chest pain patients.

One previous study reported that recent negative CIT results would predict the low risk of cardiac ischemia with a median follow-up of 35 months regardless of their initial risks of ACS [11]. Such findings might question the value of using traditional HEART scores for risk stratification, given the fact that patients with risks of ACS might already have higher HEART scores despite recent negative cardiac imaging. Since 10–20% of chest pain patients may already have the previous CIT upon presenting to the ED, the current HEART score to determine patients’ ACS risk may bring less value for their chest pain management [12, 13]. On the contrary, it may increase the providers’ burden to further admit patients to in-hospital management with the potential redundant cardiac workup. A previous study found that combining the HEART score and patient’s stress test results improved diagnostic performance of 30-day MACE outcome [14]. However, no modified HEART score was derived from the study. A similar recommendation was also shown in a recently published guideline for reasonable and appropriate care of recurrent low-risk chest pain patients in the ED (GRACE) [15]. Taken together, it might be worthwhile to modify the current HEART scoring system.

Modification and expansion of the current HEART score have been reported in previous studies [16, 17]. Some studies kept several main HEART components and replaced others [17, 18], while other studies added extra components on HEART score [14, 16]. All these studies intended to increase the diagnostic and prognostic performance accuracy of the HEART score. However, these modified scoring systems have disadvantages that prevent them from being used broadly, either limited to a subset of ED chest pain patients [18], with less commonly used variables [17], or with significant expansion of the HEART components [16], making it hard for their applications. At present, reports on suitable modification of the current HEART score are still sparse.

### Importance

An accurate scoring system will help standardize clinical practice, minimize healthcare disparity, and improve patient quality control. The HEART scoring system has been proven to risk-stratify ED chest pain patients in general successfully. However, the current HEART scoring system may still have its limitation and cannot risk-stratify accurately due to the diversity of ED chest pain patients. Since a significant number of ED chest pain patients had previous CIT findings, it is necessary to modify the current HEART scoring system by including the CIT findings to improve the performance accuracy of these patients. Meanwhile, the performance accuracy of the HEART score should maintain the same among other ED chest pain patients without the CIT findings. Thus, we believe such a modified HEART scoring system may have broader applications.

### Goal of this investigation

In this study, we aim to (1) develop a modified HEART score, HEART2, by adding the previous CIT findings as an extra scoring component to predict 30-day MACE outcome among ED chest pain patients, (2) compare the performance accuracy of using HEART versus HEART2 score for 30-day MACE outcome predictions, and (3) further determine the value of HEART2 in a subset group of ED chest pain patients—ones with previous CIT findings.

## Methods

### Study design and setting

This was a single-center retrospective study. JPS Health Network is a publicly funded tertiary referral center, which is also a level one trauma center, a chest pain center, and a comprehensive stroke center. JPS Health Network Emergency Department (ED) has annual patient volume of approximately 120,000–130,000, among which, chest pain is one of the most common chief complaints. In January 2017, we built in a HEART clinical decision tool in the electronic health record (EHR) to recommend using such a tool for risk stratification and disposition guidance. Considering the physician judgements and discretion always exceed the decision tool, using tool to determine cardiac risk and disposition was not mandatory. The HEART scores were calculated upon the patient’s presentation to the ED in the EHR. This study has been carried out in accordance with The Code of Ethics of the World Medical Association (Declaration of Helsinki) for studies involving human subjects. The regional Institutional Review Board approved this study with a full waiver of informed consent (#1541042 UNTHSC regional IRB).

### Study participants

All patients aged 18 or older who presented to the study ED with the chief complaint of chest pain or chest pain mimics (e.g., shortness of breath, dizziness, nausea, jaw pain, shoulder pain, mid-epigastric pain, etc.) between January 1, 2017, and December 31, 2019, were screened. We included all patients who had HEART score calculated with at least one EKG and one troponin test done in the ED. In our ED, we used conventional troponin I (cTn I). If multiple HEART scores reported during patients’ hospital stay, only the initial HEART score assessed by ED providers and documented on ED notes was used for data analysis. If multiple HEART scores were documented by different ED providers during patients’ ED stay, we chose the last HEART scores that documented on ED notes prior to patients’ departure from ED. We excluded patients who (1) left the ED against medical advice (AMA), eloped, or left without being seen (LWBS), (2) directly transferred to other facilities, transferred to the heart catheterization lab, or expired at the ED, and (3) who did not have one troponin tested during their indexed ED visits. In addition, all patients were expected to follow up within 30 days of discharge from the ED or hospitalization. For patients who had missed information on 30 days follow-up, imputed data were used for the analysis [12].

### Study variables

#### General variables

We collected patients’ basic demographics (i.e., age, gender, race/ethnicity) and clinical variables (i.e., patient mode of arrival at ED, insurance type, ED disposition, and total ED length of stay in minutes). A detail explanation of general variables had been reported previously [12].

#### Key variable

Cardiac imaging tests (CIT) consist of stress tests and heart catheterizations in this study. Stress tests included exercise treadmill stress test, exercise stress echocardiography, dobutamine stress echocardiography, pharmacologic nuclear Lexiscan (Regadenoson) examination, and nuclear exercise stress testing with myocardial perfusion imaging. If multiple CIT were performed in the past, the most recent ones performed were reviewed. Two independent reviewers reviewed all stress tests and heart catheterization results after completing training with cardiologist and senior emergency physicians. Briefly, Stress test results were categorized as: (1) low-risk, (2) moderate-risk, (3) high-risk, and (4) inconclusive. Positive stress test findings refer to moderate-risk and high-risk categories. Heart catheterization tests were categorized as: (1) normal/no intervention, (2) interventions, and (3) recommend further procedures. Positive heart catheterization findings refer to performing interventions or recommending further procedures. A detail description on CIT reviewing decisions were reported previously [12].

### Outcome measures

Our primary outcome is short-term (30 days) MACE outcomes. MACE referred to acute myocardial infarction (AMI), coronary revascularization by the percutaneous coronary intervention (PCI) with or without additional interventions, coronary artery bypass graft surgery (CABG), and all-cause mortality. ICD-10 code was used for determining AMI, and procedure code was used for PCI/CABG determinations. Our secondary outcome is ED disposition (e.g., discharge, admission, etc.).

Approximately 37.6% of the patients in this data sample had no follow-up information, and therefore their 30-day MACE outcomes were not observed. We thus manipulated the missing values as previously reported [12]. Briefly with three different methods: (1) All the missing MACE outcome values are indicated by zero (i.e., no MACE occurred); (2) The relevant data of patients without follow-up records were excluded from the analysis for predicting the MACE outcome; and (3) The missing MACE outcome values were imputed based on the distribution of the outcome among those follow-up patients [12].

### Development of HEART2 scoring system used in recurrent chest pain patients

Since nearly 20% (1874/9419) of chest pain patients from this study had at least one CIT performed in the past, a modified HEART scoring system has been derived. It is based on the previous results of negative CIT findings within a certain timeframe having few MACE outcomes [11, 19, 20], and modified Delphi’s techniques [21, 22]. Such a modified HEART scoring system is referred to as the HEART2 score (Table 1). Briefly, we modified the HEART score with the addition of previous CIT results (Testing), keeping all the HEART scoring components. We scored patients with the most recent negative CIT findings two years from the indexed ED visit as “− 1”. If patients’ most recent negative CIT findings were beyond two years or no CIT performed in the past, we scored as “0”. Patients with the most recent positive findings were scored as “1” regardless of the timeframe. Therefore, the total HEART2 scores ranged from − 1 to 11. To keep consistent with the HEART chest pain risk categories, we categorized HEART2 score of − 1 to 3 as low risk, 4–6 as moderate, and 7–11 as high risk.

**Table 1:** Components of HEART2 scoring system

History	Slightly suspicious	0
	Moderately suspicious	1
	Highly suspicious	2
EKG	Normal	0
	Non-specific repolarization disturbance	1
	Significant ST deviation	2
Age	< 45	0
	45–64	1
	≥ 65	2
Risk factors	No known risk factors	0
	1–2 risk factors	1
	≥ 3 risk factors or history of atherosclerotic disease	2
Troponin	≤ normal limit	0
	1–3 × normal limit	1
	≥ 3 × normal limit	2
Testing	Previous negative cardiac imaging test findings within 2 years	− 1
	Previous negative cardiac image test beyond 2 years	0
	Previous positive cardiac imaging test findings	1

### Data analysis

Data retrieval and validation was described in the previous report [12]. Descriptive analysis was conducted on patient demographics, clinical information, and primary/secondary clinical outcomes among the ED chest pain patients. Kappa statistics ($\kappa )$ were used to determine the inter-rater variability of reporting CIT results by independent reviewers with $\kappa$ > 0.8 indicating a high consistency. The HEART score was used to determine associations with the clinical outcomes. We used classification accuracy rate to determine the provider adherence of using HEART score for hospital admissions. A rate of more than 80% indicates providers’ high adherence to HEART score, while an accuracy rate of less than 50% indicates low adherence. Sensitivity, specificity, positive predictive value (PPV), and negative predictive value (NPV) were calculated to evaluate the performance accuracy of predicting MACE outcome in each patient group. We used the HEART score of 0–3 as low-risk, 4–6 as moderate-risk, and 7–10 as high-risk. The area under the curve (AUC) with its 95% confidence interval (CI) was used for the HEART score performance accuracy measure. Meanwhile, the HEART2 score was derived, and its performance accuracy of predicting MACE outcome and hospital admissions was also measured using the same analyses as mentioned above. Wilcoxon Rank-sum test was used for nonparametric continuous data comparisons (e.g., age, ED length of stay in minutes). All categorical data were compared using Pearson’s *Chi-*square test with $p$ < 0.05 as a statistically significant difference. Data analysis was conducted using STATA statistical software version 14.2 (Stata Corp, College Station, TX, USA).

### A sub-cohort analysis

Since the HEART2 score mainly affected ED chest pain patients with previous CIT findings, a sub-cohort analysis was done only on these patients. We calculated the HEART2 score on each sub-cohort chest pain patient. The performance accuracy of HEART and HEART2 scores were again compared.

### Reporting guideline

The reporting of this study conforms to the STARD statement (STAndards for Reporting Diagnostic accuracy studies) [23].

## Results

**Fig. 1:**
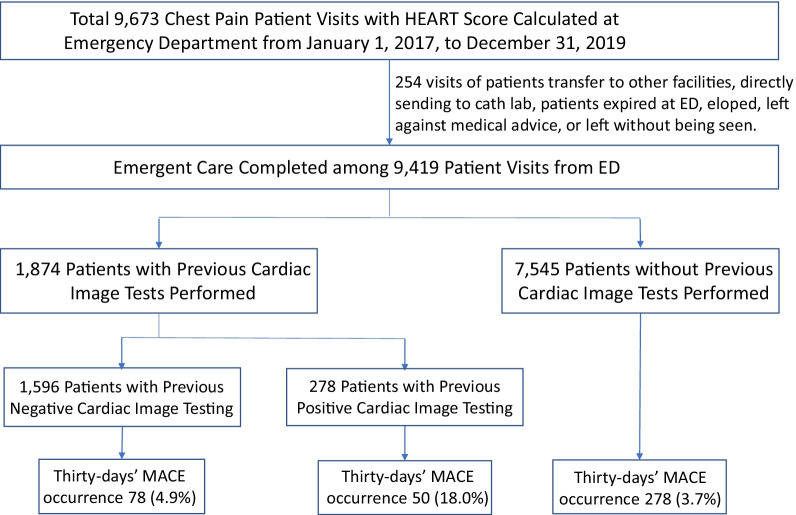
Study flow diagram

From January 1, 2017, to December 31, 2019, HEART scores were calculated prospectively among 9673 chest pain patients at the study ED. A further screening excluded patients who were transferred directly to emergent catheterization laboratories or other facilities, patients who expired at the study ED, who signed AMA, eloped, or LWBS. Thus, a total of 9419 patients were included in the final analysis (see Fig. 1). Two independent reviewers reviewed the CIT findings with high consistency ($\kappa$=0.90).

### Characteristics of study subjects

Table 2 shows the general characteristics of the study patients. In our study cohort, nearly 20% (1874/9419) of chest pain patients had previous CIT performed. Patients who had previous CIT performed tended to be older than those with no previous CIT performed (Table 2). In terms of race/ethnicity, more Non-Hispanic Black (NHB) patients included in this study than the Non-Hispanic White and Hispanic/Latino patients regardless of their CIT performed. In addition, more patients with previous CIT performed received follow-ups than those without, and they tended to arrive at ED more via medical-assisted vehicles. However, more chest pain patients with no previous CITs were self-insured than ones with previous CITs (Table 2).

**Table 2:** General characteristics of study patients

	Chest pain patients with previous CIT performed (n = 1874)	Chest pain patients without previous CIT (n = 7545)	P value
Age—year			
Mean (SD)	55.8 (10.3)	48.5 (13.1)	< 0.0001
Median (IQR)	55 (49, 63)	49 (39, 57)	< 0.0001
Gender—n (%)			
Male	924 (49.3)	3641 (48.3)	0.419
Female	950 (50.7)	3903 (51.7)	
Race/ethnicity—n (%)			
NHW	665 (35.5)	2164 (28.7)	< 0.0001
NHB	683 (36.5)	2739 (36.3)	
Hispanic/Latino	444 (23.7)	2204 (29.2)	
Others*	82 (4.4)	438 (5.8)	
Insurance—n (%)			
Hospital sponsored	544 (29.0)	1745 (23.1)	< 0.0001
Medicare	241 (12.9)	473 (6.3)	
Medicaid	65 (3.5)	234 (3.1)	
Self-pay	244 (13.0)	2638 (35.0)	
Others**	780 (41.6)	2455 (32.5)	
Mode of ED arrival—n (%)			
Medical assisted	613 (32.7)	1968 (26.1)	< 0.0001
Private	1021 (54.5)	4690 (62.2)	
Others***	240 (12.8)	887 (11.8)	
Patient follow-up, yes—n (%)	1535 (81.9)	4346 (57.6)	< 0.0001

*Race/ethnicity (others) include American Indian, Alaska Native, Asian, Native Hawaiian or Pacific Islander, or unknown, etc. **Type of insurance (others) include different commercial insurances, Tarrant County Jail, TRICARE, Cooks, Veterans insurance, and workers’ compensation insurance, etc. ***Mode of Arrival (others) include ambulatory, public transportation, taxi, police vehicle, wheelchair, or unknown

### Main results

Table 3 shows the clinical information comparison based upon patients’ previous CITs. Chest pain patients who had previous CITs tended to stay at ED longer (487 min versus 328 min, p < 0.0001) and were more likely to be placed in hospital (61.5% versus 41.8%, p < 0.0001) than ones without. Most of these patients with previous CIT performed were categorized as moderate risks using the HEART scores but recategorized as low risks by using the HEART2 scores (38.2% low risk from HEART scores vs. 55.5% low risk from HEART2 scores, p < 0.001). ED physicians had a higher consistency to place moderate-to-high HEART score patients to hospital among patients with previous CIT performed, compared to those without CIT (85.4% versus 82.7%, p = 0.015). In addition, MACE outcomes were higher among chest pain patients with the previous CIT performed than ones without (6.8% versus 3.7%, p < 0.0001).

**Table 3:** Clinical information comparisons in chest pain patients with/without previous cardiac imaging tests

	Chest pain patients with previous CIT performed (n = 1874)	Chest pain patients without previous CIT (n = 7545)	P value
ED length of stay—min			
Mean (SD)	829.0 (870.5)	652.9 (836.7)	< 0.0001
Median (IQR)	487 (272, 1210)	328 (230, 767)	< 0.0001
ED disposition—n (%)			
Discharged	721 (38.5)	4394 (58.2)	< 0.0001
Admitted	1153 (61.5)	3151 (41.8)	
HEART score—n (%)			
Low risk (0–3)	716 (38.2)	4941 (65.5)	< 0.0001
Moderate risk (4–6)	1081 (57.7)	2501 (33.2)	
High risk (7–10)	77 (4.1)	103 (1.4)	
Classification accuracy rate	85.4% (1601/1874)	82.7% (230/278)	0.015
HEART2 score—n (%)			
Low risk (–1–3)	1040 (55.5)	4941 (65.5)	< 0.0001
Moderate risk (4–6)	739 (39.4)	2501 (33.2)	
High risk (7–11)	95 (5.1)	103 (1.4)	
Time interval from previous CIT to the index ED visit—n (%)			
< 1 year	1130 (60.3)		
1–2 years	476 (25.4)		
> 2 year	268 (14.3)		
MACE outcomes—positive n (%)	128 (6.8)	278 (3.7)	< 0.0001

**Fig. 2:**
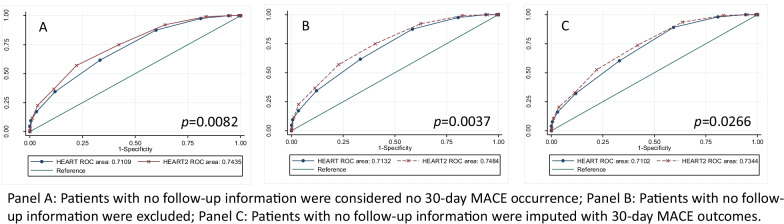
Performance accuracy comparisons between HEART and HEART2 scores among chest pain patients with previous cardiac image tests

Since switching from HEART to HEART2 score only affected patients with previous CIT performed. A sub-cohort analysis was performed mainly focused on such patients (Additional file 1: Table S1). When we compared the performance accuracy between HEART and HEART2 scores, we first considered all patients who lost follow-up as no MACE outcomes occurred within 30 days of ED discharge. We found that 55.5% of recurrent chest pain patients who used HEART2 scores can be categorized as low risk, whereas only 38.2% of such patients were categorized as low risk when HEART scores were used (Table 4). These results indicate an increased 45.5% of patients that could be discharged if the HEART2 score can be used to risk-stratify chest pain patients with previous CIT performed. More importantly, in terms of their MACE outcomes, no significant MACE outcome differences occurred among patients in all three risk groups regardless of whether HEART or HEART2 score was used (p > 0.05, Table 4). Though the sensitivity of using the HEART score predicting MACE outcomes was higher than using the HEART2 Scores (87.5% versus 75.0%), the specificity of the HEART score to predict MACE outcomes was lower (62.1% versus 57.7%). The overall performance accuracy of using the HEART2 score to risk-stratify chest pain patients was better than using the HEART score’s (AUC 0.74 versus 0.71, p = 0.0082, Table 4). Second, we only analyzed patients who had followed up within 30 days from the index ED/hospital discharge. There was a total of 1535 patients in this sub-cohort analysis. The AUC of using the HEART score to predict MACE outcomes was 0.71 (95% CI 0.67–0.76), and the AUC of using the HEART2 score to predict MACE outcomes was 0.75 (95% CI 0.71–0.79, p = 0.0037, Table 4). At last, when MACE outcomes were imputed among patients with missing follow-up information, the performance accuracy was still higher when the HEART2 score was used in comparison to the HEART score (AUC of HEART score: 0.71 (95% CI 0.67–0.75), versus AUC of HEART2 score: 0.73 (95% CI 0.70–0.77), p = 0.0266, Table 4; Fig. 2). Same performance accuracy analyses were also reported among all ED chest pain patients using HEART and HEART2 scores (see Additional file 1: Table S2).

**Table 4:** Performance accuracy comparisons between HEART and HEART2 score predicting MACE outcomes among chest pain patients with previous CIT performed

	HEART		HEART2		P value
	Number of patients	Positive MACE	Number of patients	Positive MACE	
Low-risk—n (%)	716 (38.2)	16 (2.2)	1040 (55.5)	32 (3.1)	0.3021
Moderate-risk—n (%)	1081 (57.7)	90 (8.3)	739 (39.4)	67 (9.1)	0.6103
High-risk—n (%)	77 (4.1)	22 (28.6)	95 (5.1)	29 (30.5)	0.8671
Patients with Low-risk scores for MACE outcome predictions					
Sensitivity (%, 95% CI)	87.5 (80.5–92.7)		75.0 (66.6–82.2)		
Specificity (%, 95% CI)	40.1 (37.8–42.4)		57.7 (55.4–60.1)		
PPV (%, 95% CI)	9.7 (8.0–11.5)		11.5 (9.4–13.9)		
NPV (%, 95% CI)	97.8 (96.4–98.7)		96.9 (95.797.9)		
Overall performance accuracy (AUC)					
Include missing follow-up patients	0.71 (0.67–0.75)		0.74 (0.70–0.79)		0.0082
Exclude missing follow-up patients	0.71 (0.67–0.76)		0.75 (0.71–0.79)		0.0037
Imputed missing follow-up patients	0.71 (0.67–0.75)		0.73 (0.70–0.77)		0.0266

## Discussion

Chest pain is one of the common chief complaints that present to Emergency Departments (ED) [24]. Among all chest pain patients, a significant number of patients have recurrent chest pain with frequent ED visits [15, 25]. In this study, we focused on a special population of chest pain patients who presented to the ED with previous cardiac imaging tests performed. When the HEART score was applied to such patients for cardiac risk stratification, we found that patients had higher hospitalization rates and higher 30-day MACE occurrence when compared with general ED chest pain populations. Therefore, a modified HEART2 score was derived. This modified HEART2 score improved the overall performance accuracy of predicting 30-day MACE outcomes and significantly increased the recognition of cardiac low-risk patients.

At present, ED chest pain patients with the previous CIT performed are over-investigated. Though not accounting for the majority of ED chest pain patients, such a cohort may use significant healthcare resources [15]. Frequent ED visits, higher hospital readmissions, and repetitive cardiac imaging tests significantly increased health care costs [10, 26, 27]. However, on the other hand, the impact of missing a myocardial infarction is exceptionally high, especially among US ED physicians due to the ubiquity of malpractice litigation [28]. Therefore, it is essential to choose an optimal scoring system for chest pain risk stratification. The HEART2 score derived from this study seems to be a suitable candidate based on the following reasons: (1) the short-term MACE outcome occurrence has no statistically significant difference when compared with those of the HEART score; (2) the performance accuracy of HEART2 is better than HEART among ED chest pain patients with the previous CIT performed (Table 4), and the overall performance accuracy of HEART2 is the same as HEART among all ED chest pain patients (Additional file 1: Table S2); (3) the significantly increased number of chest pain patients with previous CIT performed can be considered as a low cardiac risk category; and (4) the use of HEART2 score can be expanded to the entire ED chest pain patient population since it does not change the essential components of the traditional HEART score. Patients who have not had previous CITs would have identical scores irrespective of whether the HEART or HEART2 score is used. When HEART2 scores were used to determine ED patients disposition, if keeping the similar compliance rate of ED physicians using HEART score to stratify cardiac low-risk patients (i.e., over 80% of classification accuracy rate in this study), we would expect that an increase of nearly 50% chest pain patients with previous CITs can thus be safely discharged from ED. Those chest pain patients, even placed in a chest pain observation unit, with an average hospital cost of short stay, would probably exceed $1000/patient stay, simply switching HEART score to HEART2 score would save millions of US healthcare dollars [29, 30].

In an emergency care setting, we believe that using the HEART2 score does not burden ED physicians for patient evaluation. They would need only to incorporate patients’ previous CIT findings that have already resulted. With the broad use of electronic health records, such findings are easy to find and review. The same categories of differentiating low-risk versus moderate-to-high risk chest pain patients make it easy for ED physicians to remember. Higher HEART2 scores indicate a higher risk of short-term MACE occurrence, similar to the HEART scores [5]. The only difference is the previous CIT findings, which we consider, is one appropriate component added to the initial HEART scoring system. Previous positive CIT findings may add additional risk to chest pain patients. A study reported that patients who had stents placed often required repeated CIT to determine re-stenosis if chest pain recurred, and 30–40% new abnormal findings could thus be found with repeated CITs [31]. However, previous negative CIT findings may reduce patients’ future cardiovascular events. The WOMEN trial reported that more than 80% of patients did not require repeated cardiac testing within two years if their exercise stress test reported a low-to-intermediate probability of ischemic heart disease [19]. In another study, among patients with no history of coronary artery disease (CAD), the cardiovascular events were significantly low (< 1%) within one year if such patients had negative CIT findings [20]. We understand that negative CIT findings are not guaranteed to provide a “safe window”, different studies showed different timeframes between the initial negative cardiac imaging tests and negative cardiovascular events. One study showed that even in patients with a history of CAD, cardiovascular events were similar at one year (5.6%) and three years (6.6%) after negative CIT [20]. Most of our study patients had CIT performed within two years, especially those with previously positive CIT findings (Additional file 1: Table S1), similar to the previous reports [20]. Putting it all together, these previous study results indicate the necessity of adding previous CIT findings to the initial cardiac evaluation among ED chest pain patients.

### Limitations

First, given the nature of retrospective study design, patient selection bias, incomplete data, missing data cannot be avoided entirely, although our data were collected directly via electronic medical records. Second, the abnormal findings of CIT were determined based on charts reviewed by two independent reviewers, and even though our results reached a high level of agreement, variability may still occur. Additionally, the study hospital did not provide coronary CT angiography, therefore, such CIT was not included in this study. Third, though chest pain patients with low-risk HEART2 scores (i.e., − 1–3) can be safely discharged from ED with similar MACE occurrence in comparison to patients with low-risk HEART scores, the MACE occurrence rate may still exceed some ED physicians’ comfortable zone due to high medical-legal risks. As always, physician discretion should always exceed any scoring systems. Even with the ED discharge, such patients might still require closer clinical follow-ups. Lastly, we used this modified HEART2 score to risk-stratify low-risk recurrent chest pain patients in a single center with our unique patient population. Such findings might only be applied to this cohort. A large-scale multicenter prospective study is warranted for external validation.

## Conclusions

In conclusion, using the HEART2 score at Emergency Department might be suitable to stratify low-risk chest pain patients with previous cardiac image tests performed with a similar 30-day MACE occurrence compared to the HEART score. More importantly, when similar low-risk HEART score criteria were used on HEART2 scores, over 45% of chest pain patients with previous CITs could be discharged directly from the ED.

## Supplementary Information

**Additional file 1. Table S1**: General and Clinical Information in ED Chest Pain Patients with Previous Cardiac Imaging Tests Performed. **Table S2**. Performance accuracy comparisons between HEART and HEART2 score predicting MACE outcomes.

## Data Availability

The data that support the findings of this study are available from JPS Health Network, but restrictions apply to the availability of these data, which were used under license for the current study, and so are not publicly available. Data are however available from the authors upon reasonable request and with permission of JPS Health Network and the corresponding author.
