# Supplementary material for: Using HEART2 score to risk stratify chest pain patients in the Emergency Department: an observational study
Source: BMC Cardiovasc Disord. 2022 Mar 4;22:79. doi: 10.1186/s12872-022-02528-6 (PMC8896146; doi:10.1186/s12872-022-02528-6)
Supplement: Supplementary file 1 — Additional file 1. Table S1: General and Clinical Information in ED Chest Pain Patients with Previous Cardiac Imaging Tests Performed. Table S2. Performance accuracy comparisons between HEART and HEART2 score predicting MACE outcomes. [file 12872_2022_2528_MOESM1_ESM.docx]

Supplemental Table 1. General and Clinical Information in ED Chest Pain Patients with Previous Cardiac Imaging Tests Performed

|  | Recurrent Chest Pain Patients with Previous Negative CIT findings  (n=1,596) | Recurrent Chest Pain Patients with Previous Positive CIT findings  (n=278) | P value |
| --- | --- | --- | --- |
| Age --- year  Mean (SD)  Median (IQR) | 55 (10)  55 (49, 63) | 58 (10)  59 (52, 65) | 0.0002  <0.0001 |
| Gender --- n (%)  male  female | 768 (48)  828 (52) | 156 (56)  122 (44) | 0.014 |
| Race/Ethnicity --- n (%)  NHW  NHB  Hispanic/Latino  Others * | 527 (33)  615 (39)  385 (24)  69 (4.3) | 138 (50)  68 (24)  59 (21)  13 (4.7) | <0.0001 |
| Insurance --- n (%)  Hospital sponsored  Medicare  Medicaid  Self-pay  Others ** | 470 (29)  190 (12)  50 (3.1)  217 (14)  669 (42) | 74 (27)  51 (18)  15 (5.4)  27 (9.7)  111 (40) | 0.005 |
| Mode of ED Arrival --- n (%)  Medical Assisted  Private  Others*** | 515 (32)  878 (55)  203 (13) | 98 (35)  143 (51)  37 (13) | 0.532 |
| Patient Follow-up, yes --- n (%) | 1,301 (85) | 295 (87) | 0.288 |
| ED Length of stay --- minutes  Mean (SD)  Median (IQR) | 791 (814)  459 (268, 1,176) | 1,045 (1,119)  738 (301, 1,410) | <0.0001  0.0006 |
| ED Disposition --- n (%)  Discharged  Admitted | 673 (42)  923 (58) | 48 (17)  230 (83) | <0.0001 |
| HEART Score --- n (%)  Low risk (0-3)  Moderate risk (4-6)  High risk (7-10)  Classification Accuracy Rate | 682 (43)  864 (54)  50 (3.1)  85.9% (1,371/1,596) | 34 (12)  217 (78)  27 (9.7)  82.7% (230/278) | <0.0001  0.1677 |
| HEART2 Score --- n (%)  Low risk (-1-3)  Moderate risk (4-6)  High risk (7-11) | 1,035 (65)  543 (34)  18 (1.1) | 5 (1.8)  196 (71)  77 (28) | <0.0001 |
| Time interval from previous CIT to the index ED visit --- n (%)  <1 year  1-2 years  >2 year | 916 (57)  435 (27)  245 (15) | 214 (77)  41 (15)  23 (8.3) | <0.0001 |
| MACE outcomes --- positive n (%) | 78 (4.9) | 50 (18) | <0.0001 |

* Race/ethnicity (others) include American Indian, Alaska Native, Asian, Native Hawaiian or Pacific Islander, or unknown, etc. ** Type of insurance (others) include different commercial insurances, Tarrant County Jail, TRICARE, Cooks, Veterans insurance, and workers’ compensation insurance, etc. *** Mode of Arrival (others) include ambulatory, public transportation, taxi, police vehicle, wheelchair, or unknown.

Supplemental Table 2. Performance accuracy comparisons between HEART and HEART2 score predicting MACE outcomes

|  | HEART | | HEART2 | | P value |
| --- | --- | --- | --- | --- | --- |
|  | Number of Patients | Positive MACE | Number of Patients | Positive MACE |  |
| Low-risk --- n (%) | 5,657 (60.1) | 46 (0.8) | 5,981 (63.5) | 62 (1.0) | 0.2461 |
| Moderate-risk --- n (%) | 3,582 (38.0) | 294 (8.2) | 3,240 (34.4) | 271 (8.4) | 0.8491 |
| High-risk --- n (%) | 180 (1.9) | 66 (36.7) | 198 (2.1) | 73 (36.9) | 0.9615 |
| Patients with Low-risk scores for MACE outcome predictions |  |  |  |  |  |
| Sensitivity (%, 95% CI) | 88.7 (85.2, 91.6) | | 84.7 (80.9, 88.1) | |  |
| Specificity (%, 95% CI) | 62.3 (61.2, 63.3) | | 65.7 (64.7, 66.7) | |  |
| PPV (%, 95%CI) | 9.6 (8.7, 10.6) | | 10.0 (9.0, 11.1) | |  |
| NPV (%, 95% CI) | 99.2 (98.9, 99.4) | | 99.0 (98.7, 99.2) | |  |
| Overall performance accuracy (AUC)  Include missing follow-up patients  Exclude missing follow-up patients  Imputed missing follow-up patients | 0.82 (0.80, 0.84)  0.80 (0.78, 0.82)  0.78 (0.76, 0.80) | | 0.82 (0.80, 0.84)  0.80 (0.79, 0.82)  0.79 (0.77, 0.80) | | 0.6156  0.0846  0.3306 |
